# The Role of Electronic Medical Records in Reducing Unwarranted Clinical Variation in Acute Health Care: Systematic Review

**DOI:** 10.2196/30432

**Published:** 2021-11-17

**Authors:** Tobias Hodgson, Andrew Burton-Jones, Raelene Donovan, Clair Sullivan

**Affiliations:** 1 The University of Queensland Business School The University of Queensland St Lucia Australia; 2 Princess Alexandra Hospital Metro South Health Woolloongabba Australia; 3 The University of Queensland Centre for Health Services Research The University of Queensland Herston Australia

**Keywords:** clinical variation, unwarranted clinical variation, electronic health record, EHR, electronic medical record, EMR, PowerPlan, SmartSet, acute care, eHealth, digital health, health care, health care outcomes, outcome, review, standard of care, hospital, research, literature, variation, intervention

## Abstract

**Background:**

The use of electronic medical records (EMRs)/electronic health records (EHRs) provides potential to reduce unwarranted clinical variation and thereby improve patient health care outcomes. Minimization of unwarranted clinical variation may raise and refine the standard of patient care provided and satisfy the quadruple aim of health care.

**Objective:**

A systematic review of the impact of EMRs and specific subcomponents (PowerPlans/SmartSets) on variation in clinical care processes in hospital settings was undertaken to summarize the existing literature on the effects of EMRs on clinical variation and patient outcomes.

**Methods:**

Articles from January 2000 to November 2020 were identified through a comprehensive search that examined EMRs/EHRs and clinical variation or PowerPlans/SmartSets. Thirty-six articles met the inclusion criteria. Articles were examined for evidence for EMR-induced changes in variation and effects on health care outcomes and mapped to the quadruple aim of health care.

**Results:**

Most of the studies reported positive effects of EMR-related interventions (30/36, 83%). All of the 36 included studies discussed clinical variation, but only half measured it (18/36, 50%). Those studies that measured variation generally examined how changes to variation affected individual patient care (11/36, 31%) or costs (9/36, 25%), while other outcomes (population health and clinician experience) were seldom studied. High-quality study designs were rare.

**Conclusions:**

The literature provides some evidence that EMRs can help reduce unwarranted clinical variation and thereby improve health care outcomes. However, the evidence is surprisingly thin because of insufficient attention to the measurement of clinical variation, and to the chain of evidence from EMRs to variation in clinical practices to health care outcomes.

## Introduction

### Variation in Health Care

Any health care service seeks to raise and refine the standard of care it provides to patients and to satisfy the quadruple aim of health care, that is, to improve patient care, population health, cost of care, and clinician experience [1,2]. It is commonly accepted that achieving this aim involves minimizing unwarranted clinical variation, that is, unjustified differences between health care processes or outcomes compared with peers, or with a gold standard [3].

Health care clinical practice variation has been observed, studied, and documented for many decades [4,5]. There are a plethora of potential causes of variation, such as the individuals involved (clinician and patient), their level of agency or motivation, organizational or system factors (eg, capacity) and the nature of the evidence available (clinical and scientific) [6,7]. The method of diffusion of best practice clinical knowledge and clinician adoption of these guidelines and standards has been long been identified as a potential cause of variation [8,9].

Many countries mandate efforts to reduce unwarranted clinical variation in health care provided [10]. While some level of variation is required for innovation and learning, low levels of variation are generally thought to be best [11]. As stated in [12], “The idea is to hold on to variation across patients (to meet the needs of individual patients) and to limit variation across clinicians (which is driven by individual clinician preferences or differences in knowledge and experience)”.

Variation is unwarranted if it is not justified by clinical imperatives, patient needs or preferences, or innovation. In its most basic form, clinical variation that leads to positive outcomes may be warranted, whereas variation that leads to negative outcomes is deemed unwarranted. Many health care services have looked to electronic medical record (EMR) systems to reduce unwarranted variation and thereby improve outcomes [10].

### EMRs

EMR use has become virtually ubiquitous in health services in developed countries [13]. EMRs can offer many benefits, including improvements in billing and cost management, reporting and analytics, real-time access to data by clinicians, information sharing, treatment management, patient safety, and clinical decision making [14-21].

EMRs provide the means to both monitor and address clinical variation through the provision of best practice guidelines and clinical decision support (CDS) to improve care and reduce waste [22]. At the same time, EMRs can also create variation by offering users multiple ways to perform a task. Work-as-done by clinicians also often varies from the work-as-imagined expectation of EMR designers [23]; as a result, it is an empirical question as to whether EMRs actually reduce unwarranted clinical variation.

### Theoretical Framework

Studying how EMRs may affect unwarranted clinical variation requires understanding 3 elements: why clinical variation occurs, why and how EMRs may reduce clinical variation, and how measuring and altering variation are operationalized in practice (Textbox 1).

**Clinical variation factors. CDS: clinical decision support, EMR: electronic medical record.**

- Clinical variation can occur due to supply-side, demand-side, or contextual factors [24]:
  - Clinician factors (supply side): expertise, training and experience, preference, practice style;
  - Consumer factors (demand side): case complexity, consumer preference, social determinants of health; and
  - Environmental factors (context): local guidelines, available resources, hospital case mix.
- EMRs may reduce clinical variation through their ability to control process delivery and outcomes. It is common for health services to tackle clinical variation through EMR-related process control efforts (eg, clinical guidelines and pathways), and process design and development efforts [25,26]. EMRs hold promise for reducing unwarranted clinical variation because they can help tackle each of the 3 aforementioned factors:
  - Clinician factors: EMRs can constrain clinicians to perform similarly via restrictions to particular behaviors or range of behaviors.
  - Consumer factors: EMRs can inform and guide patients in a consistent manner via patient portals, and they can help standardize the reporting of patient outcomes.
  - Environmental factors: EMRs can provide standardized decision support and data that health services can use to monitor and improve operations and achieve greater consistency.
- Understanding precisely how an EMR can reduce unwarranted variation requires opening the EMR “black box” and assessing its components. One set of EMR components designed to help reduce unwarranted clinical variation is CDS. There are numerous CDS tools and features in the marketplace, with EMR vendors naming and implementing components in proprietary ways. This review focuses on 2 CDS components from the 2 most prevalent EMR vendors globally (>50% of acute care market), with products that have similar aims to help reduce unwarranted clinical variation: PowerPlan (Cerner Corporation) and SmartSet (Epic Systems Corporation) [27-29]:
  - PowerPlan: “A Power Plan is a group of orders under a single title designed to support a procedure or a process.” [30]
  - SmartSet: “A documentation template. A group of orders and other elements, such as notes, chief complaints, SmartGroup Panels, and levels of service, that are commonly used together to document a specific type of visit.” [31].

EMRs can implement tools to guide and constrain practice; however, clinicians do not always use these interventions as intended. For example, they may focus on using a PowerPlan to make ordering easier rather than using it to reduce variation. For this reason, it is important to empirically test whether in practice they reduce clinical variation as intended.

**Figure 1:**
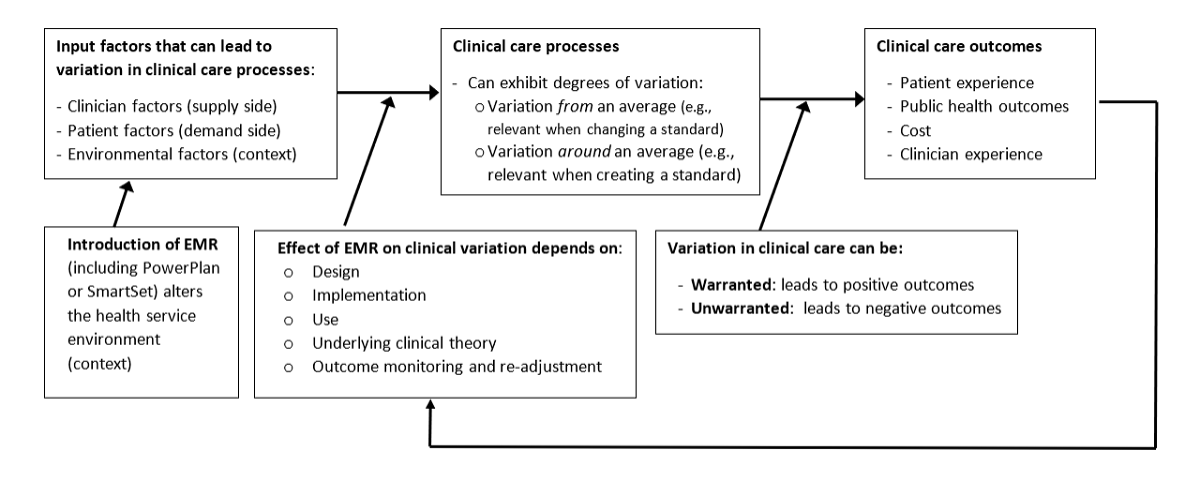
Variation in clinical care - theoretical framework. EMR: electronic medical record.

To understand how EMR interventions might or might not reduce unwarranted clinical variation as intended, a variation in clinical care framework was devised (Figure 1). The framework highlights the expected factors that must be accounted for if EMRs are to reduce unwarranted clinical variation. That is, the expectation is that EMRs—through their components—should help reduce unwarranted clinical variation if the following factors are considered:

- *Design:* if the EMR and its PowerPlan or SmartSet components are configured to reduce unwarranted variation.
- *Implementation:* if the goal of reducing unwarranted variation is kept in focus during implementation.
- *Use:* if clinicians use the EMR as intended.
- *Clinical theory:* if the clinical logic or theory underlying the design of the intervention and the clinical practice is mature (rather than lacking evidence and having ambiguity, allowing variation among clinicians).
- *Monitoring and intervention:* if the health service monitors outcomes that flow from changes in clinical variation and iteratively improve the design and use of the EMR based on this learning feedback loop.

If the use of an EMR can lead to changes in unwarranted clinical variation, how can this variation be measured? The framework (Figure 1) suggests that there are 2 archetypal changes in variation (Textbox 2), conveying different meanings of “variance.”

Combinations of these 2 archetypes may also occur. For instance, a health service may implement an EMR to both change a standard and encourage clinicians to achieve greater consistency around that standard.

**Changes in variation.**

- *Variation from a level or a standard:* For example, assume that a health service has a guideline for a clinical practice. If clinicians follow the guideline, with appropriate variation in adherence and excellent outcomes, this will be reflected in an average level on that practice with variation around the average. If the health service shifts the guideline, unwarranted variation can be viewed as the degree to which the distribution of behavior fails to shift to the new standard and improve outcomes. Statistically, this can be tested by comparing the average practices (accounting for the variation around each average) before and after the intervention, (eg, via a *t* test).
- *Variation around a level or a standard:* For example, assume that a health service has no guideline for a practice, and clinicians just follow their own practices. Assume also that the average behavior is close to the desired level, but the variation around this average is concerning. If the health service then implements a guideline to reduce this variation, unwarranted clinical variation and monitoring of outcomes can be operationalized as the degree to which the level of variance in practices fails to be reduced. Statistically, this can be tested by a change in the level of variance (eg, range or SD).

The implication of these different meanings of clinical variation is that researchers need to be precise as to which type of variation and associated outcomes they are studying and how. In short, studying changes in variation requires careful attention to measurement.

Finally, variation is only unwarranted if it impairs outcomes, such as any of the quadruple aims of health care (Figure 1). That is, variance itself is not the outcome, nor it is necessarily negative. Rather, the aim is to learn how to design, implement, use, and monitor the EMR and find the “right” level of variation to achieve the best outcomes.

### Objective of This Review

We aim to summarize the existing literature on the effects of EMRs on variation in clinical care processes and patient outcomes as mapped to the quadruple aim of health care. To account for the specifics of EMR systems, and for the specific ways that variation can occur, searches were conducted not only for the effects of EMRs in general, but also for the components of EMRs (PowerPlan and SmartSet). Studies were coded for changes in clinical variation and for how changes in variance affected both process and patient outcomes.

Because of differences in tools and methods used to achieve clinical standardization between the primary and acute care settings (eg, case complexity, technology utilized), this study focuses purely on the acute sector and hospital-based EMRs.

## Methods

### Eligibility Criteria

A Preferred Reporting Items for Systematic Reviews and Meta-Analyses (PRISMA)–compliant systematic review of studies examining clinical variation and EMRs was undertaken [32].

#### Inclusion Criteria

To be included in the review, studies were required to meet the following criteria:

- The article is published in English.
- The topic of the study is relevant to clinical variation and EMRs.
- Articles published after 2000, due to the prominence of EMR/electronic health record (EHR) articles published since then (Multimedia Appendix 1).
- Participants to include clinicians performing medical care duties or patients receiving medical treatment.
- Measured outcomes were reported, whether immediate (eg, test results) or longer term (eg, length of stay, economic), and whether measured objectively or by self-report.
- Peer-reviewed studies only.
- Empirical studies are either qualitative or quantitative (or mixed): quantitative studies may have included experimental and observational study designs such as randomized controlled trials, quasi-experimental studies, before-and-after studies, case–control studies, cohort studies, and cross-sectional studies.
- Quality improvement initiatives.
- Articles focused on acute care settings (including ambulatory specialist care).

#### Exclusion Criteria

Studies were excluded from the review if they met any of the following criteria:

- Abstracts in which full study data were unavailable.
- Nonempirical studies.
- Outcome measures of expected variation (not actual).
- Articles with a care focus of primary care.

### Information Sources

Searches were made on ACM Digital Library, CINAHL, EMBASE/MEDLINE, Google Scholar, IEEE Xplore, PubMed, Scopus, and Web of Science for articles from the year 2000 to November 2020.

The search query used was: “EHR” OR “EMR” AND “practice variation” OR “clinical variation” OR “unwanted variation” OR “unwarranted variation” OR “reduction in waste” OR “PowerPlan” OR “SmartSet”.

As noted earlier, understanding how EMRs have their effects requires opening the “black box” of the EMR to study its components, in this case PowerPlan and SmartSet. However, a given study may use these proprietary terms or instead use more generic terms. Including these specific vendor EMR components in the search string with an OR term increased the extent to which articles that examined clinical variation, even if an article did not specifically use those words (ie, to increase the level of recall), would be found.

Additional applicable search terms were assessed but excluded as they added no additional search results (eg, medical-order entry systems). The term “order sets” was excluded from the search as they are not necessarily electronic (often paper based) and many studies focus on discrete point-in-time events (eg, prescribing anithrombotics) rather the patient’s entire care process (as implemented in PowerPlan and SmartSet for specific conditions). As noted earlier, the focus of this study was on clinical care processes and outcomes.

The ultimate searches were undertaken in February 2021. Both backward and forward citation searching were undertaken for all included articles with a quality score over 50% (35/36 studies; Multimedia Appendix 2) [33-67]. Forward searches were undertaken with the assistance of Anne O’Tate, PubMed, Google Scholar, and Scopus [68].

Once duplicates were removed, these searches resulted in 4622 potential articles. Titles and abstracts were then identified and screened, with 3935 initial further exclusions, with 40 cases having only partial text available or requiring further information to make an assessment, resulting in 687 full texts that were retrieved and evaluated.

Interrater agreement during the screening phase was assessed based on 30 randomly sampled papers screened by 2 reviewers (TH [first author] and TL [research assistant]). The observed agreement was 90% (27/30), with an acceptable κ (Cohen κ) of 0.67 [69]. Given the reliable coding, the remainder of the screening phase was undertaken by TH.

**Figure 2:**
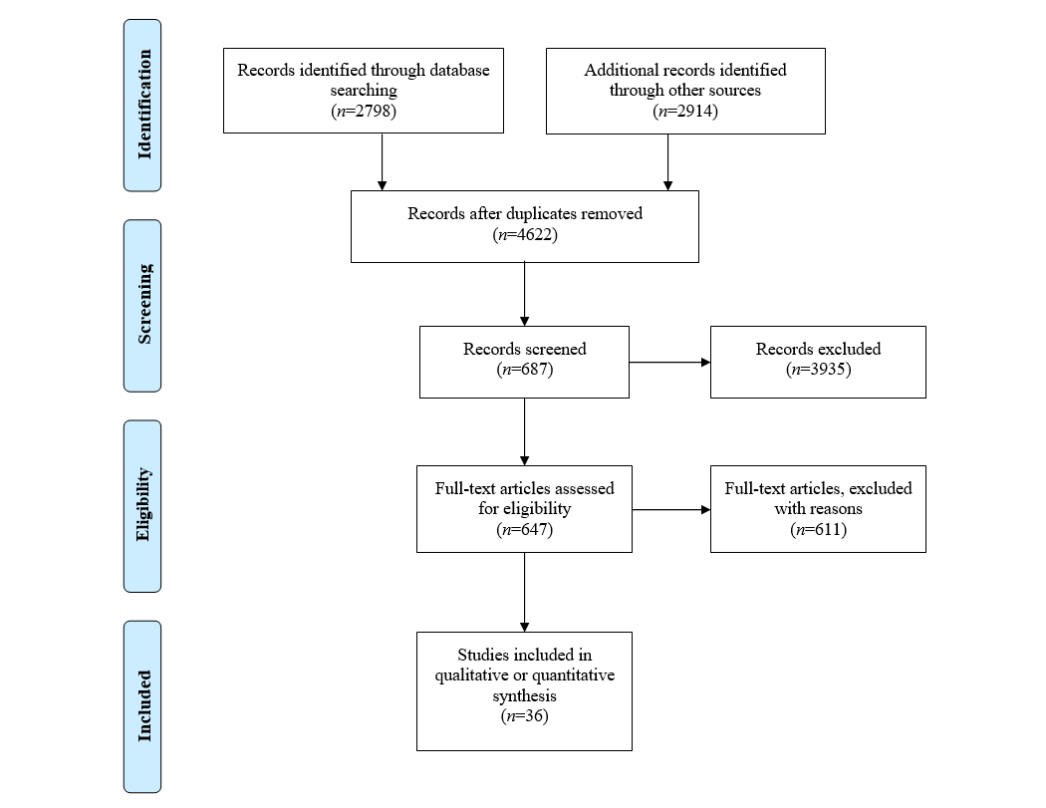
Systematic review flow diagram (after Preferred Reporting Items for Systematic Reviews and Meta-Analyses [PRISMA] [32]).

Each of the included articles was assessed independently by 2 reviewers (TH and TL) against the inclusion criteria. After assessment, 36 studies remained [33-67,70] (Figure 2) (Table 1). In instances of doubtful eligibility, a consensus assignment was made after deliberation (5 articles were excluded). The 2 reviewers also measured the disposition of these studies as positive, mixed, or negative based on how the authors of the study discussed the outcomes.

**Table 1:** Summary of included studies (N=36).

Study author	EMR^a^/EHR^b^ vendor	Study disposition	Quality assessment (QATSDD^c^), %	Variation in clinical care processes	Variance type^d^	Patient care^e^	Population health^e^	Costs/ efficiency^e^	Clinician experience^e^
Adelson et al [33]	EPIC (SmartSet)	Positive	54.17	Orders/prescription	2	Clinical events		Length of events	Quality (clinician)
Akenroye et al [34]	Vendor not stated	Positive	64.58	Orders/prescription	2			Costs	Quality (clinician)
Amland et al [35]	Cerner (PowerPlan)	Positive	78.57	Patient assessment	2	Clinical events			
Asan et al [36]	EPIC (SmartSet)	Negative	66.67	Care provision	5				Clinical burden
Attaar et al [37]	Other: Allscripts Sunrise Clinical Manager	Positive	66.67	Orders/prescription	4	Quality (patient)		Length of stay	
Ballesca et al [38]	EPIC (SmartSet)	Positive	66.67	Orders/prescription	2	Clinical events Test measures		Length of stay	
Borok et al [39]	EPIC (SmartSet)	Positive	61.90	Orders/prescription Referrals	4				Clinical burden
Bradywood et al [40]	Vendor not stated	Positive	80.95	Clinical care pathway	4	Quality (Patient) Clinical events		Length of stay Length of events	
Chisolm et al [41]	Vendor not stated	Positive	77.08	Orders/prescription	5			Costs Length of stay	Quality (clinician)
Dort et al [42]	Vendor not stated	Positive	73.81	Clinical care pathway	5	Clinical events		Length of stay Length of events	
Ebinger et al [43]	Vendor not stated	Positive	66.67	Care provision	4	Clinical events		Costs Length of stay	
Geltman et al [44]	Vendor not stated	Mixed	71.43	Patient assessment	2	Test measures			
Goga et al [45]	Vendor not stated	Positive	54.76	Orders/prescription	4				
Gulati et al [46]	Cerner (PowerPlan)	Positive	76.19	Orders/prescription	2	Clinical events		Length of stay Length of events	
Hendrickson et al [47]	Vendor not stated	Positive	78.57	Orders/prescription	2	Clinical events		Number of tests	
Hooper et al [48]	Vendor not stated	Positive	66.67	Patient assessment	2	Test measures			
Horton et al [49]	EPIC (SmartSet)	Positive	59.52	Orders/prescription	2	Quality (patient) Clinical events		Test measures	
Jacobs et al [50]	Other: ICIS, a web-based EHR	Positive	71.43	Ordering	2				
Karajgikar et al [51]	Cerner (PowerPlan)	Positive	54.76	Orders/prescription	5	Clinical events		Length of events Length of stay	
Kicker et al [67]	Vendor not stated	Positive	57.14	Ordering Preparation	2			Costs Length of events	
Lewin et al [52]	Vendor not stated	Positive	59.52	Orders/prescription Use of intervention	4			Costs Length of stay	
Lindberg et al [53]	EPIC (SmartSet)	Positive	76.19	Patient assessment	2	Test levels			
Lindberg et al [54]	EPIC (SmartSet)	Positive	73.81	Patient assessment	5	Test levels			
Morrisette et al [55]	Cerner (PowerPlan)	Mixed	69.05	Ordering	4			Costs Length of events	
Prevedello et al [56]	Other: Percipio; Medicalis Corp	Mixed	73.81	Patient assessment	2			Test measures	
Reynolds et al [57]	EPIC (SmartSet)	Negative	61.90	Orders/prescription	4				
Rooholamini et al [58]	Cerner (PowerPlan)	Positive	59.52	Orders/prescription Patient assessment	2	Clinical events		Costs Length of events	
Rosovsky et al [70]	EPIC (SmartSet)	Positive	45.24	Ordering	4				
Sim et al [59]	Other: AllScripts	Positive	69.05	Ordering	2				
Sonstein et al [60]	EPIC (SmartSet)	Positive	69.05	Ordering	4	Clinical events		Length of stay	
Soo et al [61]	Cerner (PowerPlan)	Negative	68.75	Ordering	4			Length of events	Clinical burden
Studer et al [65]	Vendor not stated	Positive	61.90	Orders/prescription	2	Clinical events			
Teich et al [66]	Vendor not stated	Positive	42.86	Ordering	2				
Terasaki et al [62]	EPIC (SmartSet)	Positive	64.29	Patient assessment	2				
Wang et al [63]	EPIC (SmartSet)	Positive	52.38	Orders/prescription	4	Quality (patient)		Volume of drugs	
Webber et al [64]	Cerner (PowerPlan)	Positive	57.14	Ordering	4			Costs	

^a^EMR: electronic medical record.

^b^EHR: electronic health record.

^c^QATSDD: Quality Assessment Tool for Studies with Diverse Designs.

^d^1=Mean constant; variance change, 2=mean change; variance change, 3=mean change; variance constant, 4=mean change; variance unknown, 5=mean unknown; variance unknown (or N/A, assumed only).

^e^Where the outcomes were not observed within the study table cells remain empty.

Study data including the intervention, population, study design, and effects were extracted by both reviewers using a standardized template within Covidence systematic review software (Multimedia Appendix 3) [71]. Data quality was assessed via a bespoke Covidence template employing the Quality Assessment Tool for Studies with Diverse Designs (QATSDD), a 16-item mixed methods quality assessment tool (Multimedia Appendix 2) [72].

### Risk of Bias

The studies were examined to determine the risk of drawing biased inferences [73]. Five risks were identified (Textbox 3).

**Risk of bias. EMR: electronic medical record.**

1. Publication bias: most papers (30/36, 83%) reported positive results [33-35,37-43,45-54,58-60,62-66,70], with a minority reporting mixed [44,55,56] or negative results [36,57,61] (3/36, 8% for both). The completeness of results including nonsignificant effects was not always assured.
2. Selection: participation in the trials varied from compulsory to voluntary. Where the study was voluntary, it was more likely that those with interest in, and with a positive opinion toward, EMRs participated [36,41,57,58,67].
3. Randomization of intervention: this only occurred in 1 study which randomized the use of the SmartSet intervention using block randomization, stratified by provider subspecialty [57].
4. Performance: the studies were all composed of unblinded trials, and in many cases the participants of the study knew if they were utilizing the intervention or not.
5. Time lag bias: some papers were reporting on data collected much earlier than publication date (eg, Teich et al [66] was based on 1993 data) [41,66].

### Recruitment

The recruitment of participants for clinicians utilizing the interventions was voluntary in all but 2 studies, and existing clinic/hospital EMR data were utilized for patient data [33,55].

### Coding

**Figure 3:**
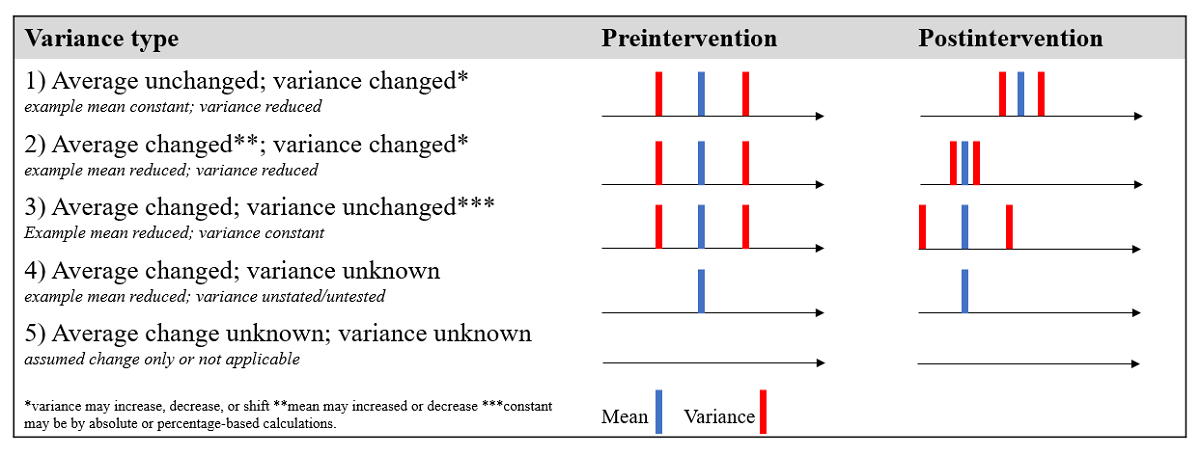
How changes in variance can be operationalized in clinical practice.

Following the earlier description of how variation in clinical practices can be observed, studies were coded for 5 types of variation, each reflecting different patterns in the change of a distribution (Figure 3 and Multimedia Appendix 3). Types 1 and 3 refer to the 2 archetypes noted earlier (“variance from” and “variance around”), whereas Type 2 reflects their combination. Type 4 reflects the possibility that a study refers to changes in average behavior without reporting changes in variance. Type 5 is where change is assumed but not measured.

As this study’s aim is to learn the effects of the EMR on changes in clinical variation, the focus is on variance types 1, 2, and 3, which reflect different ways in which clinical variation can be expressed. By contrast, Types 4 and 5 do not provide clear measures of variation.

To code the study’s disposition, 2 reviewers (TH and TL) coded the overall disposition of a study as either positive, mixed, or negative, based on the following criteria:

- Positive: a majority of studies stated expected outcomes were met.
- Mixed: some elements of expected outcomes were met, some not (with an approximate 50/50 split).
- Negative: intervention not used, majority of expected outcomes not met, or reverse outcomes seen.

Disposition reflects the authors’ overall conclusions in that study in favor of or against the EMR or the intervention. It is not a measure of whether a study measured clinical variation or outcomes. Interrater agreement on study disposition was calculated using Cohen κ, and showed high levels of agreement (33/36, 92%, κ=0.71) [69].

Clinical outcomes were coded according to the quadruple aim of health care: quality of patient care, population health, cost/efficiency, and clinician experience [1,2].

## Results

Almost all the studies were based on the implementation of an intervention (new or refined) into a clinical setting (35/36, 97%) with 1 qualitative analysis of EMRs by clinicians [36]. Most studies were quality or process improvement based (28/36, 78%) [33-35,37,39-45,47-49,51,52,54,55,58,59,61-67] or best practice/evidence-based intervention related (27/36, 75% for both) [33-35,37,38,40-42,45,47,48,50,52-60,62-66,70]. Over half of the studies examined EMR elements such as order sets (23/36, 64%) [33,34,36-38,40-42,46,47,49-51,52,54,55, 58,60,61,64-66,70] and care pathways/treatment plans (22/36, 61%) [33-36,39-43,46-48,50,52,54,58,60,62,63,65,66,70]. Many papers addressed the minimization or elimination of a particular drug prescription/use (17/36, 47%) [39,40,45,46,49,51-54,57, 58,60,63,65-67,70].

Of the papers where the specific EMR used by the health facility was identified (24/36, 67%), half were Epic (12/24, 50%) [33,36,38,39,49,53,54,57,59,60,62,63], some Cerner (7/24, 29%) [35,46,51,55,58,61,64], and few with other vendors (5/24, 21%) [37,50,52,56,59].

Regarding overall disposition, most studies reported positive results (30/36, 83%) [33-35,37-43,45-54,58-60,62-67,70], while a minority reported mixed [44,55,56] or negative results [36,57,61] (3/36, 8% each). That is, the authors concluded in most studies that the EMR was used successfully as part of an initiative to address clinical variation.

However, most studies did not measure or report variation. Of the 5 codes for coding variance (Figure 3), no studies reported Type 1 or 3, half reported Type 2 (18/36, 50%) [33-35,38,44,46-50,53,56,58,59,62,65-67], some reported Type 4 (13/36, 36%) [37,39,40,43,45,52,55,57,60,61,63,64,70], and a few reported Type 5 (5/36, 14%) [36,41,42,51,54]. The studies that reported results for variation coded as Types 2 and 4 generally examined how an intervention led to changes in the average of a clinical behavior. Such studies reflected Type 2 variation if they explicitly referred to measures of variance in addition to average practices or if the distribution of the variable examined was such that a change in the average clearly implied a change in variance (the dependency between the average and variance of a distribution is dependent on the type of distribution).

For example, if clinician behaviors were coded in a study as adhering or not adhering to a guideline, the rate of adherence would reflect a binomial distribution and so an increase in adherence (eg, from 60% to 80%), implying both an increase in the average behavior and a reduction in variance. Where this connection between a change in a behavior and the change in variance was not explicitly reported or could not be inferred clearly from the distribution, this reflected a Type 4 change. That is, the 13 studies coded as Type 4 found that the EMR affected clinical practices but not necessarily clinical variation.

Regarding the quadruple aims of health care outcomes, over half of the studies addressed individual care outcomes (19/36, 53%) [33,35,37,38,40,42-44,46-49,51,53,54,58,60,63,65], many examined efficiency (21/36, 58%) [33,34,37,38, 40-43,46,47,49,51,52,55,56,58,60,61,63,64,67], a handful examined clinician experience (6/36, 17%) [33,34,36,39,41,61], and none examined population health outcomes (Table 1). Some studies examined just 1 quadruple aim of health care outcome (13/36, 36%) [35,36,39,44,48,52-56,64,65,67], most studies examined 2 outcomes (15/36, 42%) [34,37,38,40-43, 46,47,49,51,58,60,61,63], 1 study examined 3 outcomes [33], and none of the studies examined all 4 outcomes associated with the widely accepted quadruple aims of health care.

Of the studies that measured changes in variation (18/36, 50%), many (11/18, 61%) [33,35,38,44,46-49,53,58,65] examined follow-on changes in clinical-care outcomes, half assessed cost outcomes (9/18, 50%) [33,34,38,46,47,49,56,58,67], few examined clinician experience outcomes (2/18, 11%) [33,34], and none addressed public health outcomes. In other words, even though studies generally reported positive findings (in terms of overall study disposition), this positive conclusion was based on a partial (rather than comprehensive) assessment of outcomes.

There was heterogeneity in study data quality, with QATSDD scores ranging from a low of 43% through to a high of 81% and a mean of 65% across all included studies (Multimedia Appendix 2).

## Discussion

### Principal Findings

This review finds some evidence to justify that EMRs can help reduce unwanted clinical variation and thereby improve health care outcomes. The evidence, however, is not strong. This reflects that (1) study quality was not high, (2) not many studies examined the effect, and (3) clinical variation and outcomes were not examined consistently (different outcome measures across studies) or comprehensively (rarely studying more than 1 outcome).

Surprisingly, while all the studies retrieved by our search discussed clinical variation, few studies measured it, and even fewer tied these changes in clinical variation to a broad set of health care outcome measures.

The theoretical framework proposed earlier can be used to understand the results of the review and identify directions for research. Specifically, 5 factors can enhance the EMR’s effects on unwarranted clinical variation and follow-on health care outcomes: design, implementation, use, clinical theory, and outcome monitoring and re-adjustment (Figure 1). These factors were examined only sporadically across studies with an average of 3 addressed per paper, and only 4 of the 36 retrieved studies examined all 5 factors [34,41,43,49].

### Design

Intervention design was discussed in most studies (27/36, 75%) [33,34,36,37,40-50,53-55,58,59,61-65,67,70] but not in depth. While not a core focus of the studies, design-related issues that may affect clinical variation were identified, such as in the insights that “design characteristics that are intended to make documentation more efficient can have unintended consequences” and that “some of the suboptimal design characteristics of the EHR may be exacerbated by user-related practices.” [36].

### Implementation

Almost all the studies (35/36, 97%) [33-35,37-67,70] examined the implementation of a new or refined intervention into a clinical setting, but specific implementation details were found in fewer studies (23/36, 64%) [33-35,37,40-46, 48-50,52,55,56,59-62,64,70]. The introduction of EMRs and their components are in large part a change management process, with both situational and psychological aspects to consider [74]. The successful implementation of change requires the participation, commitment, and support of key organizational stakeholders throughout the life span of the process to provide the highest chance of success [75,76].

### Use

One way to improve outcomes is to educate and train users to employ the EMR more effectively. The role of education and training was addressed in the majority of studies (25/36, 69%) [34,37,39-41,43,44,46-49,52-58,60-64,66,70] and frequently mentioned as critical for the intervention’s success or failure. Education/training was also identified as requiring primary focus in those studies deemed as having a negative or mixed disposition [36,44,55-57,59]. A multifaceted approach with local super-user support, high-quality training materials, and education and feedback sessions is likely to help. For instance, a 2018 study by Robinson [77] of Kaiser Permanente saw a significant increase in the use of many order sets after the implementation of a 3-day intensive EMR education intervention specifically tailored for the physicians with interactive teaching methods.

### Underlying Clinical Theory

The interventions in the retrieved studies were all developed on underlying clinical theory that explicitly or implicitly directs clinical practice via pathway, program, or guidelines. These varied from locally developed standards established from journal articles and consensus guidelines, or more commonly the implementation of established national or peak body guidelines. Given that clinical care should be tailored to the needs of patients in the local setting, how best to identify and customize the appropriate underlying theory for a guideline and how stringently to implement it in the EMR are open questions that require further research.

### Outcome Monitoring and Re-adjustment

Only 10 studies addressed monitoring of clinical outcomes and re-adjustment of the interventions. Even when addressed, they were typically confined to the implementation phase, rather than long-term and ongoing monitoring and revisions. Using EMRs to implement feedback loops and quality management life cycles can help health care organizations improve safety and quality and become learning organizations [78]. Intermountain Healthcare has shown how this can be achieved via repeated cycles of *create*, *distribute*, *use*, *monitor*, and *feedback* [79,80].

### Limitations

Despite steps taken to perform high-quality searching, sample bias may still exist. Because this is an understudied topic, the required search terms and meta-tags on the topic are not yet mature and validated. As a result, different search terms could potentially have retrieved additional relevant publications. Gray literature (such as internal health service reports) may also exist on the topics that were not retrieved. The time span of included studies was broad, covering over 20 years, but a longer time span may have identified additional papers.

Differences in the design and scope of the retrieved papers prevented direct comparisons among studies and meta-analytic tests. Judgment also needed to be exercised when coding articles. While interrater reliability tests suggested that the coding was reliable, some subjectivity inevitably remained. Finally, the context faced by a health service (eg, its resources and patient mix) influences how an EMR can help. Given the small number of studies in this area and their heterogeneity, it was not possible to pinpoint the most salient elements of context.

Expanding the study to include nonacute health care settings, articles in languages other than English, and specifying additional EMR vendors may provide valuable insight into additional means and methods available to address EMR-based clinical variation beyond those identified within this review.

### Comparison With Prior Work

Existing studies and reviews on comparable topics were examined and while there is much existing work addressing the effects of EMRs on health care quality and outcomes, and measuring various criteria (efficiency, guideline adherence, errors, clinical outcomes), none adequately or directly address these aspects through the lens of clinical variation and outcomes [81,82]. No previous studies related variation and clinical outcomes back to the quadruple aims of health care. The ability to map variation in EMR-related clinical care processes and outcomes to all 4 of the quadruple aims (patient experience, public health, cost, and clinician experience) sets this review apart from any prior work in the field (Figure 1).

### Conclusions

EMRs and their components such as PowerPlans/SmartSets are not a panacea, but rather tools to assist health care provision. It is widely thought that evidence-based clinical guidelines play an essential role in promoting quality of care and minimizing unwanted variation [83]. Ideally, EMRs should be able to improve both the average clinical practices and reduce unwarranted variation. However, the effects of unwarranted variation on clinical outcomes are unclear and understudied.

This review finds some evidence to suggest that unwarranted variation can be reduced, but the evidence is not strong. Many studies focused on technical outcomes (eg, adoption, reduction in variation), rather than on the clinical health care outcomes themselves. More research is needed to learn how EMRs can be implemented and used to reduce unwarranted variation; however, it is important to remember that reduction in clinical variation itself is not the desired outcome. Rather, improved health care outcomes are the ultimate goal.

It is critical that these health care outcomes are clearly defined and monitored, in concert with the ongoing reduction in variation driven by EMRs as a mechanism, to create a continuous learning health care system with appropriate governance to keep iteratively improving health care outcomes over time.

### Recommendations

Additional empirical research on EMRs and how their elements such as PowerPlans/SmartSets affect clinical variation and patient outcomes is needed. More attention needs to be given on how to: (1) measure clinical variation and unwarranted variation; (2) improve the effects of an EMR on reducing unwarranted clinical variation; (3) measure multiple elements of the quadruple aim of health care in a single study; and (4) articulate and test the chain of evidence from the EMR to changes in clinical variation to outcomes.

## Appendix

Multimedia Appendix 1 Electronic medical record/electronic health record prevalence in published studies figure.

Multimedia Appendix 2 Quality assessment summary table using the Quality Assessment Tool for Studies with Diverse Designs (QATSDD).

Multimedia Appendix 3 Data extraction coding table.

## Abbreviations

CDS: clinical decision support
EHR: electronic health record
EMR: electronic medical record
PRISMA: Preferred Reporting Items for Systematic Reviews and Meta-Analyses
QATSDD: Quality Assessment Tool for Studies with Diverse Designs
