# Supplementary figures and images for: The Role of Electronic Medical Records in Reducing Unwarranted Clinical Variation in Acute Health Care: Systematic Review
Source: JMIR Med Inform. 2021 Nov 17;9(11):e30432. doi: 10.2196/30432 (PMC8663492; doi:10.2196/30432)

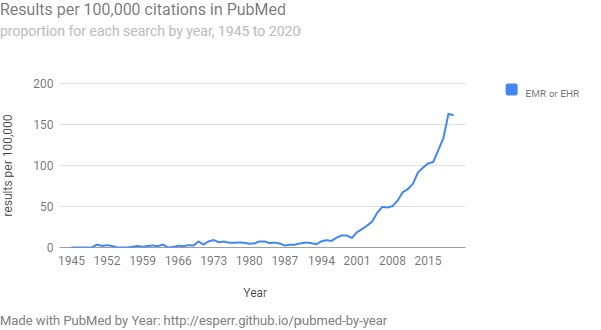

Supplement: Multimedia Appendix 1 [file medinform_v9i11e30432_app1.png]
