# Supplementary material for: The Role of Electronic Medical Records in Reducing Unwarranted Clinical Variation in Acute Health Care: Systematic Review
Source: JMIR Med Inform. 2021 Nov 17;9(11):e30432. doi: 10.2196/30432 (PMC8663492; doi:10.2196/30432)
Supplement: Multimedia Appendix 2 [file medinform_v9i11e30432_app2.docx]

## Appendix 2: QATSDD quality assessment summary table

| **Study ID** | **Data Quality** | **Q1)** | **Q2)** | **Q3)** | **Q4)** | **Q5)** | **Q6** | **Q7)** | **Q8)** | **Q9)** | **Q10)** | **Q11)** | **Q12)** | **Q13)** | **Q14)** | **Q15)** | **Q16)** |
| --- | --- | --- | --- | --- | --- | --- | --- | --- | --- | --- | --- | --- | --- | --- | --- | --- | --- |
| Adelson 2014 | 54.17% | 1 | 3 | 2 | 2 | 2 | 2 | 1 | 1 | 2 | 2 | 1 | 1 | 2 | 1 | 3 | 0 |
| Akenroye 2016 | 64.58% | 1 | 3 | 3 | 0 | 2 | 2 | 2 | 2 | 2 | 2 | 2 | 2 | 2 | 2 | 3 | 1 |
| Amland 2014 | 78.57% | 2 | 3 | 3 | 2 | 2 | 3 | 2 | 2 | 2 | 2 |  | 2 | 2 |  | 3 | 3 |
| Asan 2018 | 66.67% | 2 | 3 | 3 | 2 | 2 | 2 | 2 | 2 |  |  | 2 | 2 | 1 | 2 | 2 | 1 |
| Attaar 2020 | 66.67% | 2 | 3 | 2 | 1 | 2 | 2 | 2 | 1 | 2 | 2 |  | 2 | 2 |  | 2 | 3 |
| Ballesca 2014 | 66.67% | 2 | 2 | 2 | 2 | 2 | 2 | 2 | 2 | 3 | 2 |  | 2 | 2 |  | 1 | 2 |
| Borok 2018 | 61.90% | 1 | 3 | 3 | 2 | 2 | 1 | 1 | 1 | 3 | 2 |  | 3 | 1 |  | 2 | 1 |
| Bradywood 2017 | 80.95% | 1 | 3 | 3 | 2 | 2 | 3 | 3 | 2 | 2 | 3 |  | 3 | 2 |  | 2 | 3 |
| Chisolm 2006 | 77.08% | 2 | 3 | 3 | 2 | 2 | 2 | 2 | 3 | 2 | 2 | 2 | 2 | 3 | 2 | 2 | 3 |
| Dort 2020 | 73.81% | 2 | 3 | 2 | 2 | 3 | 3 | 3 | 1 | 2 | 2 |  | 2 | 3 |  | 1 | 2 |
| Ebinger 2016 | 66.67% | 1 | 3 | 3 | 1 | 1 | 2 | 2 | 2 | 2 | 2 |  | 2 | 2 |  | 3 | 2 |
| Geltman 2015 | 71.43% | 2 | 3 | 3 | 1 | 2 | 2 | 2 | 1 | 2 | 3 |  | 2 | 3 |  | 1 | 3 |
| Goga 2017 | 54.76% | 2 | 3 | 3 | 1 | 1 | 1 | 1 | 1 | 2 | 2 |  | 2 | 1 |  | 1 | 2 |
| Gulati 2018 | 76.19% | 2 | 3 | 3 | 2 | 2 | 2 | 2 | 1 | 3 | 3 |  | 3 | 2 |  | 1 | 3 |
| Hendrickson 2018 | 78.57% | 2 | 2 | 3 | 2 | 2 | 2 | 3 | 2 | 3 | 2 |  | 2 | 2 |  | 3 | 3 |
| Hooper 2013 | 66.67% | 2 | 2 | 3 | 2 | 2 | 1 | 2 | 2 | 2 | 2 |  | 2 | 2 |  | 2 | 2 |
| Horton 2020 | 59.52% | 2 | 2 | 2 | 1 | 2 | 2 | 2 | 2 | 2 | 2 |  | 2 | 2 |  | 1 | 1 |
| Jacobs 2012 | 71.43% | 2 | 3 | 3 | 2 | 2 | 2 | 2 | 1 | 2 | 2 |  | 2 | 2 |  | 2 | 3 |
| Karajgikar 2018 | 54.76% | 1 | 2 | 2 | 1 | 2 | 2 | 1 | 1 | 2 | 2 |  | 2 | 2 |  | 1 | 2 |
| Kicker 2018 | 57.14% | 2 | 3 | 3 | 1 | 1 | 1 | 2 | 2 | 2 | 2 |  | 1 | 1 |  | 1 | 2 |
| Lewin 2019 | 59.52% | 1 | 2 | 2 | 1 | 1 | 2 | 2 | 2 | 2 | 2 |  | 2 | 1 |  | 2 | 3 |
| Lindberg 2014 | 76.19% | 2 | 3 | 3 | 2 | 2 | 3 | 2 | 2 | 2 | 2 |  | 2 | 2 |  | 2 | 3 |
| Lindberg 2016 | 73.81% | 2 | 3 | 3 | 2 | 2 | 2 | 2 | 2 | 2 | 2 |  | 2 | 2 |  | 2 | 3 |
| Morrisette 2015 | 69.05% | 2 | 3 | 3 | 2 | 1 | 2 | 2 | 2 | 2 | 2 |  | 2 | 2 |  | 2 | 2 |
| Prevedello 2013 | 73.81% | 2 | 3 | 3 | 2 | 2 | 2 | 2 | 2 | 2 | 2 |  | 2 | 2 |  | 2 | 3 |
| Reynolds 2020 | 61.90% | 2 | 2 | 2 | 2 | 2 | 2 | 2 | 2 | 2 | 2 |  | 2 | 1 |  | 1 | 2 |
| Rooholamini 2017 | 59.52% | 2 | 2 | 3 | 2 | 1 | 1 | 1 | 2 | 2 | 2 |  | 2 | 1 |  | 2 | 2 |
| Rosovsky 2020 | 45.24% | 2 | 2 | 3 | 0 | 0 | 1 | 1 | 2 | 1 | 2 |  | 2 | 1 |  | 2 | 0 |
| Sim 2017 | 69.05% | 2 | 2 | 3 | 2 | 3 | 2 | 2 | 2 | 2 | 2 |  | 2 | 2 |  | 1 | 2 |
| Sonstein 2014 | 69.05% | 2 | 2 | 3 | 2 | 2 | 2 | 2 | 2 | 2 | 2 |  | 2 | 2 |  | 2 | 2 |
| Soo 2019 | 68.75% | 2 | 2 | 2 | 3 | 2 | 2 | 2 | 2 | 2 | 2 | 2 | 2 | 1 | 2 | 3 | 2 |
| Studer 2020 | 61.90% | 2 | 3 | 3 | 1 | 1 | 2 | 2 | 2 | 2 | 2 |  | 2 | 1 |  | 2 | 1 |
| Teich 2000 | 42.86% | 1 | 2 | 2 | 1 | 1 | 1 | 1 | 2 | 2 | 1 |  | 1 | 1 |  | 1 | 1 |
| Terasaki 2015 | 64.29% | 2 | 2 | 3 | 2 | 2 | 1 | 2 | 2 | 2 | 2 |  | 2 | 1 |  | 2 | 2 |
| Wang 2020 | 52.38% | 2 | 2 | 2 | 0 | 1 | 2 | 2 | 2 | 1 | 2 |  | 1 | 1 |  | 2 | 2 |
| Webber 2013 | 57.14% | 2 | 2 | 2 | 1 | 2 | 2 | 2 | 2 | 1 | 2 |  | 1 | 1 |  | 2 | 2 |

Questions:
1) Explicit theoretical framework
2) Statement of aims/objectives in main body of report
3) Clear description of research setting
4) Evidence of sample size considered in terms of analysis
5) Representative sample of target group of a reasonable size
6) Description of procedure for data collection
7) Rationale for choice of data collection tool(s)
8) Detailed recruitment data
9) Statistical assessment of reliability and validity of measurement tool(s) (Quantitative only)
10) Fit between stated research question and method of data collection (Quantitative only)
11) Fit between stated research question and format and content of data collection tool e.g. interview schedule (Qualitative only)
12) Fit between research question and method of analysis
13) Good justification for analytical method selected
14) Assessment of reliability of analytical process (Qualitative only)
15) Evidence of user involvement in design
16) Strengths and limitations critically discussed
