# Supplementary material for: The Role of Electronic Medical Records in Reducing Unwarranted Clinical Variation in Acute Health Care: Systematic Review
Source: JMIR Med Inform. 2021 Nov 17;9(11):e30432. doi: 10.2196/30432 (PMC8663492; doi:10.2196/30432)
Supplement: Multimedia Appendix 3 [file medinform_v9i11e30432_app3.docx]

## Appendix 3: Data extraction coding table

| **Coding classification** |
| --- |
| **General information** |
| Study ID |
| Title |
| Lead author contact details |
| Country in which study occurred |
| Publication type |
| Place of publication |
| Date of publication |
| **Characteristics of study** |
| Aim of study |
| Conclusion of study |
| *Method* |
| Study design |
| Study self-reported design type |
| Study type |
| Study start date |
| Study end date |
| Blinding details |
| Randomization details |
| Ethics and governance details |
| **Participants** |
| Population description |
| Total number of participants |
| Meets inclusion/exclusion criteria |
| Method of recruitment of participants |
| **EHR/EMR** |
| EMR/EHR vendor |
| Study relates to the use/modification of an EMR/EHR |
| **Themes** |
| Implementation study |
| Care pathway / care plan / treatment plan / clinical pathway (explicit/stated) |
| Order set (explicit/stated) |
| Quality/process improvement (explicit/stated) |
| Best practice / evidence based (explicit/stated) |
| Education / training (explicit/stated) |
| Alerts (explicit/stated) |
| Coding (explicit/stated) |
| Economic element/impact |
| **Outcomes/Interventions** |
| Variation description |
| Independent variables (study manipulated or measured |
| Dependent variables (potential change) |
| Outcomes measured |
| **Area of medicine** |
| Setting/area of medicine |
| Tertiary care |
| **Limitations of study** |
| Limitations addressed |
| Study funding |
| Conflict of interest |
| **Overall study inclusion/exclusion** |
| Inclusion/exclusion |
